# Predicting metabolizable energy from digestible energy for growing and finishing beef cattle and relationships to the prediction of methane

**DOI:** 10.1093/jas/skac013

**Published:** 2022-01-16

**Authors:** Kristin E Hales, Carley A Coppin, Zachary K Smith, Zach S McDaniel, Luis O Tedeschi, N Andy Cole, Michael L Galyean

**Affiliations:** 1 Department of Animal and Food Sciences, Texas Tech University, Lubbock, TX 79409, USA; 2 Department of Animal Science, South Dakota State University, Brookings, SD 57007, USA; 3 Department of Animal Science, Texas A&M University, College Station, TX 77843-2471, USA; 4 Conservation and Production Research Laboratory, USDA-ARS, Bushland, TX 79012, USA; 5 Department of Veterinary Sciences, Texas Tech University, Lubbock, TX 79409, USA

**Keywords:** beef cattle, digestible energy, metabolizable energy, methane prediction

## Abstract

Reliable predictions of metabolizable energy (**ME**) from digestible energy (**DE**) are necessary to prescribe nutrient requirements of beef cattle accurately. A previously developed database that included 87 treatment means from 23 respiration calorimetry studies has been updated to evaluate the efficiency of converting DE to ME by adding 47 treatment means from 11 additional studies. Diets were fed to growing-finishing cattle under individual feeding conditions. A citation-adjusted linear regression equation was developed where dietary ME concentration (Mcal/kg of dry matter [**DM**]) was the dependent variable and dietary DE concentration (Mcal/kg) was the independent variable: ME = 1.0001 × DE – 0.3926; *r*^2^ = 0.99, root mean square prediction error [**RMSPE**] = 0.04, and *P* < 0.01 for the intercept and slope. The slope did not differ from unity (95% CI = 0.936 to 1.065); therefore, the intercept (95% CI = −0.567 to −0.218) defines the value of ME predicted from DE. For practical use, we recommend ME = DE – 0.39. Based on the relationship between DE and ME, we calculated the citation-adjusted loss of methane, which yielded a value of 0.2433 Mcal/kg of dry matter intake (**DMI**; SE = 0.0134). This value was also adjusted for the effects of DMI above maintenance, yielding a citation-adjusted relationship: CH_4_, Mcal/kg = 0.3344 – 0.05639 × multiple of maintenance; *r*^2^ = 0.536, RMSPE = 0.0245, and *P* < 0.01 for the intercept and slope. Both the 0.2433 value and the result of the intake-adjusted equation can be multiplied by DMI to yield an estimate of methane production. These two approaches were evaluated using a second, independent database comprising 129 data points from 29 published studies. Four equations in the literature that used DMI or intake energy to predict methane production also were evaluated with the second database. The mean bias was substantially greater for the two new equations, but slope bias was substantially less than noted for the other DMI-based equations. Our results suggest that ME for growing and finishing cattle can be predicted from DE across a wide range of diets, cattle types, and intake levels by simply subtracting a constant from DE. Mean bias associated with our two new methane emission equations suggests that further research is needed to determine whether coefficients to predict methane from DMI could be developed for specific diet types, levels of DMI relative to body weight, or other variables that affect the emission of methane.

## Introduction

Predicting metabolizable energy (**ME**) from digestible energy (**DE**) is not a new concept. The *Nutrient Requirements of Farm Livestock No. 2 Ruminants* (ARC, 1965) noted that ME could be calculated from DE using a factor of 0.82. Later, the use of DE × 0.82 was adopted in the fifth, sixth, and seventh revised editions of the National Research Council (**NRC**)—*Nutrient Requirements of Beef Cattle* (NRC, 1976, 1984, 2000). Although the seventh revised edition of the NRC (2000) incorporated the 0.82 conversion, it cautioned that the ratio could vary considerably depending on intake, age of the animal, and feed source. The eighth revised edition (NASEM, 2016) reported the value of ME = 0.82 × DE (NRC, 1976; Garrett, 1980), although Vermorel and Bickel (1980) indicated that the ME:DE ratio ranged from 0.82 to 0.93 in growing cattle. Recent data indicate that the conversion of DE to ME is more efficient than previously reported, especially for cattle consuming high-concentrate diets (Hales et al., 2012, 2013, 2014, 2015a, 2015b, 2017; Fuller et al., 2020).

A reliable prediction of ME from DE is necessary because most feed NE values in current use are calculated from ME using the cubic equations developed by Garrett (1980). These equations were based on the conversion efficiency of DE to ME using a factor of 0.82. If ME values are underestimated, specifically for high-concentrate diets, net energy for maintenance (NE_m_) and retained energy (**NE**_**g**_) requirements might also be affected. Based on an analysis of literature data, Galyean et al. (2016) suggested a linear regression equation for predicting ME from DE. Our objective was to add new data to the Galyean et al. (2016) database and reevaluate their proposed equation. In addition, we describe and evaluate new methane prediction equations derived from the DE:ME relationship.

## Materials and Methods

Data used in this paper were generated from published literature; thus, no live animals were used by the authors, and Institutional Animal Care and Use Approval was not necessary.

### Statistical analyses of the DE:ME relationship

Galyean et al. (2016) used 87 treatment means from 23 papers published from 1975 to 2015 to evaluate the relationship between DE and ME. An additional 47 treatment means from 11 papers published from 2015 to 2020 were added to the original database (134 total observations). Adding these studies addressed a weakness in the original database related to a limited number of data points from lower DE (e.g., higher-forage diets). The additional studies decreased the mean DE concentration from 3.15 Mcal/kg reported by Galyean et al. (2016) to 3.05 Mcal/kg in the updated database. New papers added to the database were from experiments using growing bulls, steers, or heifers and open-circuit respiration calorimetry systems of either a chamber or a headbox, which are the same types of animals and methods that were included in the original database. Dietary DE concentrations (1.84 to 3.88 Mcal/kg), crude protein (**CP**; 7.88% to 24.08%), neutral detergent fiber (**NDF**; 15.65% to 68.81%), ether extract (**EE**; 1.94% to 8.71%), and starch concentrations (0% to 56.85%) in the added studies were either those reported in the papers or those calculated from various sources as described by Galyean et al. (2016). Dietary gross energy (GE), DE, and ME concentrations as well as energy in methane and urine were experimentally determined for each treatment mean. Methane and urine energy concentrations were calculated as a proportion of GE and DE. A brief description of the additional studies is provided in Table 1, and the complete updated database in spreadsheet format is available as Supplementary Material.

**Table 1.** Descriptive statistics for the studies added to the Galyean et al. (2016) database used for model development

Source	Diet	Animal	No. of observations	Mean BW^1^, kg	DMI, kg/d	Percentage of DM					Mcal/kg of DM			ME:DE	Percentage of DE	
						CP	NDF	Ether extract	Starch	TDN	GE	DE	ME		CH_4_	Urine
Baber et al. (2020)	Concentrate-based diet—day 116 of gestation	MARC III pregnant heifer	7	432	4.58	14.60	26.60	3.73	54.32	78.8	4.21	3.21	2.79	0.8707	7.5	6.1
Baber et al. (2020)	Concentrate-based diet—day 172 of gestation	MARC III pregnant heifer	7	468	5.13	14.60	26.60	3.73	54.32	78.8	4.11	2.94	2.55	0.8675	8.6	5.3
Baber et al. (2020)	Concentrate-based diet—day 235 of gestation	MARC III pregnant heifer	7	520	6.04	14.60	26.60	3.73	54.32	78.8	4.22	3.21	2.80	0.8711	7.7	4.6
Baber et al. (2020)	Forage-based diet—day 116 of gestation	MARC III pregnant heifer	7	430	6.70	15.10	48.70	2.11	7.49	57.8	4.25	2.48	2.04	0.8253	10.2	7.2
Baber et al. (2020)	Forage-based diet—day 172 of gestation	MARC III pregnant heifer	7	454	7.04	15.10	48.70	2.11	7.49	57.8	4.20	2.39	1.97	0.8274	10.7	6.5
Baber et al. (2020)	Forage-based diet—day 235 of gestation	MARC III pregnant heifer	7	498	8.27	15.10	48.70	2.11	7.49	57.8	4.35	2.43	2.02	0.8308	10.9	6.0
Crossland et al. (2018)	Control main effect	British crossbred steers	8	480	6.77	11.80	21.70	3.50	49.20	79.4	4.16	3.06	2.80	0.9150	3.3	4.3
Crossland et al. (2018)	Yeast main effect	British crossbred steers	8	484	6.77	11.80	21.70	3.50	49.20	79.4	4.16	3.16	2.93	0.9272	3.0	4.2
Crossland et al. (2018)	Thermoneutral main effect	British crossbred steers	8	483	7.10	11.80	21.70	3.50	49.20	79.4	4.16	3.06	2.80	0.9150	2.8	4.2
Crossland et al. (2018)	Heat stressed—main effect	British crossbred steers	8	480	6.43	11.80	21.70	3.50	49.20	79.4	4.16	3.16	2.92	0.9241	3.6	4.3
Fuller et al. (2020)	0% dry-rolled corn diet	Angus yearling steers	10	495	7.54	11.99	40.60	3.73	21.10	63.9	4.24	2.67	2.33	0.8727	8.1	4.5
Fuller et al. (2020)	22.5% dry-rolled corn diet	Angus yearling steers	10	515	8.05	12.62	39.31	3.37	24.20	68.3	4.22	2.69	2.31	0.8587	10.4	4.1
Fuller et al. (2020)	45% dry-rolled corn diet	Angus yearling steers	10	507	8.65	12.61	35.15	3.15	26.72	72.8	4.27	2.80	2.43	0.8679	9.5	3.8
Fuller et al. (2020)	67.5% dry-rolled corn diet	Angus yearling steers	10	521	7.78	12.82	28.23	3.02	36.46	78.0	4.22	2.90	2.58	0.8897	7.6	3.7
Fuller et al. (2020)	83.8% dry-rolled corn diet	Angus yearling steers	10	526	8.52	12.49	27.95	2.90	45.26	83.2	4.29	3.15	2.89	0.9175	5.1	3.0
Hales et al. (2017)	Dry-rolled corn control	Angus steers	8	475	7.23	15.02	13.87	3.00	56.85	82.5	4.40	3.22	3.01	0.9343	4.6	2.0
Hales et al. (2017)	Dry-rolled corn + 2% corn oil	Angus steers	8	471	7.32	15.02	13.46	5.61	54.50	84.3	4.54	3.31	3.11	0.9409	4.1	1.7
Hales et al. (2017)	Dry-rolled corn + 4% corn oil	Angus steers	8	481	6.99	15.02	13.23	7.72	52.60	86.1	4.67	3.34	3.16	0.9465	3.3	2.0
Hales et al. (2017)	Dry-rolled corn + 6% corn oil	Angus steers	8	489	6.90	15.09	13.23	8.71	51.45	87.9	4.80	3.44	3.27	0.9515	3.1	1.8
Hemphill et al. (2018)	Corn stalk diet—day 14	MARC III heifers	8	448	6.01	8.63	66.81	2.34	4.11	57.1	3.96	1.84	1.39	0.7545	14.4	10.1
Hemphill et al. (2018)	Monensin corn stalk diet—day 14	MARC III heifers	8	486	5.95	8.63	66.81	2.34	4.11	57.1	3.95	1.84	1.41	0.7673	13.7	9.5
Hemphill et al. (2018)	Corn stalk diet—day 42	MARC III heifers	8	457	5.30	8.63	66.81	2.34	4.11	57.1	3.91	1.86	1.39	0.7465	15.5	9.9
Hemphill et al. (2018)	Monensin corn stalk diet—day 42	MARC III heifers	8	488	5.59	8.63	66.81	2.34	4.11	57.1	3.91	1.96	1.53	0.7794	14.1	8.1
Hemphill et al. (2018)	Corn stalk diet—day 161	MARC III heifers	8	525	8.22	8.63	66.81	2.34	4.11	57.1	3.96	2.08	1.71	0.8195	10.3	8.5
Hemphill et al. (2018)	Monensin corn stalk diet—day 161	MARC III heifers	8	556	8.04	8.63	66.81	2.34	4.11	57.1	3.97	2.06	1.67	0.8119	10.6	8.1
Jennings et al. (2018)	Steam-flaked corn diet—1× maintenance	Angus cross steers	12	262	2.29	13.80	19.80	4.60	49.90	88.4	4.41	3.65	3.19	0.8736	7.7	5.1
Jennings et al. (2018)	Steam-flaked corn diet + excess CP—1× maintenance	Angus cross steers	12	261	2.30	19.50	19.40	3.50	44.80	87.5	4.53	3.86	3.32	0.8615	7.6	6.5
Jennings et al. (2018)	Steam-flaked corn diet—2× maintenance	Angus cross steers	12	391	6.15	13.80	19.80	4.60	49.90	88.4	4.41	3.73	3.42	0.9173	4.7	3.6
Jennings et al. (2018)	Steam-flaked corn diet + excess CP—2× maintenance	Angus cross steers	12	391	6.23	19.50	19.40	3.50	44.80	87.5	4.53	3.88	3.55	0.9148	4.1	4.4
Kongphitee et al. (2018)	10% Cassava pulp diet	Native Thai beef cattle	6	148	2.74	9.90	63.20	5.90	8.83	59.3	4.13	2.65	2.32	0.8750	10.5	2.3
Kongphitee et al. (2018)	30% Cassava pulp diet	Native Thai beef cattle	6	134	2.75	9.70	53.60	5.90	20.31	65.6	4.25	3.02	2.72	0.9014	8.1	1.4
Kongphitee et al. (2018)	50% Cassava pulp diet	Native Thai beef cattle	6	138	3.01	9.70	45.20	5.90	31.80	72.0	4.24	3.22	2.97	0.9211	6.9	1.0
Shreck et al. (2017)	Control—no supplement	British crossbred steers	6	212	4.79	27.23	18.87	2.28	0	69.5	3.73	2.85	2.48	0.8702	8.9	4.3
Shreck et al. (2017)	Steam-flaked corn + monensin supplement	British crossbred steers	6	214	4.50	24.08	16.58	2.30	12.36	70.0	3.00	3.00	2.64	0.8808	7.9	4.1
Tangjitwattanachai et al. (2015)	1.1× maintenance	Native Thai beef cattle	5	269	3.50	10.60	36.30	3.50	30.90	69.3	4.10	2.99	2.51	0.8395	13.7	11.5
Tangjitwattanachai et al. (2015)	1.1× maintenance	Native Thai beef cattle	5	288	4.90	10.60	36.30	3.50	30.90	69.3	4.24	3.01	2.56	0.8505	12.2	10.8
Tangjitwattanachai et al. (2015)	1.1× maintenance	Native Thai beef cattle	5	324	5.50	10.60	36.30	3.50	30.90	69.3	4.21	3.07	2.64	0.8599	11.6	10.1
Walter et al. (2016)	Control	Beef steers	10	449	3.47	13.84	15.65	6.32	53.30	88.9	4.81	3.81	3.69	0.9685	2.5	0.7
Walter et al. (2016)	Zilpaterol	Beef steers	10	455	3.47	13.84	15.65	6.32	53.30	88.9	4.80	3.87	3.72	0.9612	3.2	0.6
Wei et al. (2018)	Control corn silage diet	Chinese indigenous Wandong bulls	4	273	5.10	11.10	50.50	3.18	37.37	71.9	4.19	2.77	2.36	0.8497	11.0	4.0
Wei et al. (2018)	Control with 10% rice straw replacement	Chinese indigenous Wandong bulls	4	273	5.10	10.71	52.50	3.00	33.60	68.9	4.16	2.68	2.30	0.8566	11.0	3.4
Wei et al. (2018)	Control with 30% rice straw replacement	Chinese indigenous Wandong bulls	4	273	5.10	9.91	56.30	2.65	26.14	62.8	4.09	2.43	2.04	0.8382	11.3	5.0
Wei et al. (2018)	Control with 60% rice straw replacement	Chinese indigenous Wandong bulls	4	273	5.10	8.71	62.10	2.31	14.91	53.8	3.98	2.02	1.68	0.8306	12.1	4.7
Wei et al. (2018)	Control corn silage diet	Chinese indigenous Wandong bulls	4	276	5.10	11.10	50.50	3.18	37.37	71.9	4.19	2.69	2.31	0.8571	10.9	3.4
Wei et al. (2018)	Control with 10% wheat straw replacement	Chinese indigenous Wandong bulls	4	276	5.10	10.57	53.50	2.97	33.60	69.6	4.18	2.66	2.26	0.8519	11.2	3.7
Wei et al. (2018)	Control with 30% wheat straw replacement	Chinese indigenous Wandong bulls	4	276	5.10	9.49	59.30	2.56	26.14	65.0	4.15	2.40	2.03	0.8460	11.7	4.0
Wei et al. (2018)	Control with 60% wheat straw replacement	Chinese indigenous Wandong bulls	4	276	5.10	7.88	68.10	1.94	14.91	58.2	4.11	2.01	1.68	0.8364	12.0	4.4

BW, body weight; CP, crude protein; DE, digestible energy; DMI, dry matter intake; ME, metabolizable energy; NDF, neutral detergent fiber.

Mixed-model methods described by Littell et al. (2006) were used to evaluate the relationship between dietary DE and ME concentration. Dietary ME concentration was the dependent variable and was regressed on dietary DE concentration to evaluate the linear regression (equation 1). Study citation was included in the model as a random effect to account for variation from differing slopes and intercepts in the published studies. Citation-adjusted data were created for each data point from the simple linear model (Galyean and Tedeschi, 2014). The coefficient of determination (*r*^2^) and root mean square prediction error (**RMSPE**) were determined for the model using the citation-adjusted values and PROC MIXED and PROC REG of SAS (SAS Inst. Inc., Cary, NC; version 9.3). The coefficient of determination was used to determine the precision, and the RMSPE was used to assess model accuracy.

### Methane prediction equations

Based on the relationship between DE and ME (discussed in a subsequent section), two equations were developed to predict methane production. First, a citation-adjusted daily emission of methane (Mcal/kg of dry matter intake [**DMI**]) was determined by mixed-model regression by fitting a model with a random intercept term but no slope. Subsequently, this citation-adjusted intercept term was corrected for multiples of net energy intake required for maintenance to yield a second equation for predicting methane. The adjustment involved calculating the Mcal of NE_m_ required using metabolic body weight (**BW**^0.75^) and a NE_m_ requirement of 0.077 Mcal/BW^0.75^ (NASEM, 2016) along with the cubic equations for calculating dietary NE_m_ concentration from ME reported by Galyean et al. (2016).

These new prediction equations were evaluated and compared with other published equations using a second literature-derived database independent of the 34-study database used for equation development described above. The independent database consisted of 129 data points from 29 published studies. The studies used growing and finishing steers and heifers, in addition to five treatments means from lactating heifers. Methane losses were measured using open-circuit respiration calorimetry with either headboxes or chambers. Most of the citations included intake energy, but it was not reported in 22% of citations and was calculated according to NASEM (2016) using dietary composition. For citations that did not provide complete data, tabular values for feed ingredients (NASEM, 2016) and feed ingredient composition data were used to estimate aspects of dietary composition. Tabular calculations of this type were performed for organic matter (29% of the data), CP (19%), NDF (27%), acid detergent fiber (**ADF**; 31%), EE (57%), and starch (64%) concentrations. Most citations included either DE or ME or both. All citations included methane losses; however, only 47% of the citations reported urinary energy loss. Previously published equations and our two new equations were evaluated using this independent database by regressing observed methane on the predicted methane for each equation. In addition to the coefficient of determination and RMSPE statistics, the concordance correlation coefficient (**CCC**) was computed as described by Lin (1989), and the mean squared prediction error (**MSPE**) was decomposed by determining the mean, slope, and error biases and expressing these values as a percentage of the MSPE (Tedeschi, 2006).

## Results and Discussion

### Predicting ME from DE

**Figure 1.**
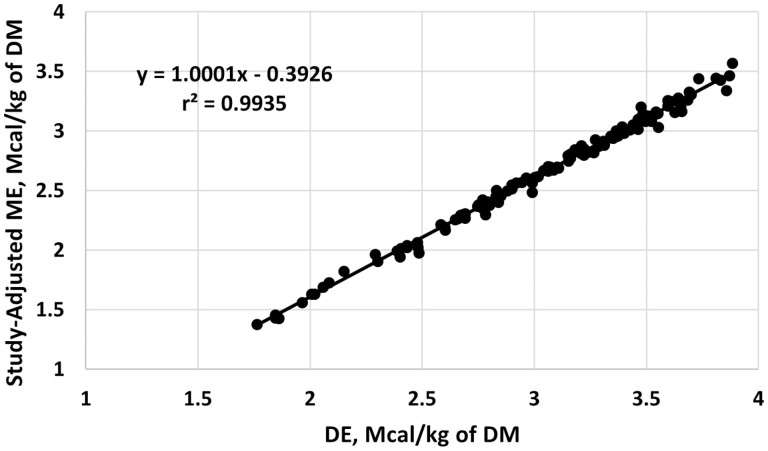
Relationship between digestible energy (DE) and metabolizable energy (ME) concentrations in the Galyean et al. (2016) database plus an additional 47 treatment means from 11 studies, adjusted for random differences in intercepts and slopes among citations (study-adjusted). The solid line is the study-adjusted regression equation, and dots are individual treatment mean observations.


The citation-adjusted linear regression equation with dietary ME concentration as the dependent variable and dietary DE concentration as the independent variable (Figure 1) was:

$$\begin{array}{l} ME=1.0001\times DE-0.3926; \end{array}$$ (1)

where ME and DE are expressed as Mcal/kg of DM ($r^{2}=\;0.994$, RMSPE = 0.0399, and *P* < 0.001 for the intercept and slope; 95% CIs: intercept [−0.567, −0.218] and slope [0.936, 1.065]).

Given that the slope of equation 1 does not differ from unity, the intercept defines the ME value predicted from DE. Thus, for routine applications, we recommend the following equation:


$$\begin{array}{l} ME=DE-0.39 \end{array}$$ (2)

Galyean et al. (2016) reported that  ME=0.9611×DE−0.2999 for growing/finishing cattle. It should be noted that the slope estimate in the Galyean et al. (2016) study also did not differ from unity (95% confidence limits were 0.9015 and 1.0207), but the authors chose to include the slope in their recommended equation.

For lactating dairy cows, Moe and Tyrrell (1977) suggested that ME=1.01×DE−0.45. The slope and intercept estimates for the Moe and Tyrrell (1977) equation are contained within the 95% confidence limits of equation 1. The Moe and Tyrrell (1977) equation was based on data from dairy cows fed at 3-times maintenance, leading the NRC (2001) to caution that the equation might not be accurate for intakes near maintenance. In the literature data used to derive equation 1, DMI levels were generally much less than those of Moe and Tyrrell (1977), ranging from 0.77% to 2.44% of mean BW (mean 1.67% [SD 0.36]), and thus more appropriate for beef cattle throughout various production stages. 

A quadratic equation for predicting ME from dietary DE concentration ($ME=-0.057\times DE^{2}+1.3764\times DE-0.9483$) was developed by Hales (2019) using individual animal observations from diets varying in forage and grain concentrate levels. When a residual analysis was conducted, the residuals from high-forage (>65% of DM) diets differed from 0, and the residuals from high-concentrate (>65 % of DM) diets did not, suggesting that the quadratic equation was not accurate in high-forage diets and should only be used in high-concentrate diets. While having prediction equations for specific diet types could be helpful, the ability to predict ME across a wide range of diets is important for estimating net energy and prescribing nutrient requirements across varying production systems.

Fuller et al. (2020) reported that a static ratio-based conversion factor for calculating ME from DE would fail to describe the biology associated with methane and urinary energy losses across a wide range in dietary DE and levels of DMI and suggested that the true relationship between DE and ME was not constant. Growing cattle were fed five diets with increasing concentrations of dry-rolled corn replacing alfalfa hay and corn silage, resulting in differing forage-to-concentrate ratios (Fuller et al., 2020). As the forage-to-concentrate ratio decreased, the conversion of DE to ME increased from 0.87 to 0.92, largely because of a quadratic response of methane energy loss and a linear decrease in urinary energy loss. Thus, Fuller et al. (2020) concluded that the ME:DE ratio should be expressed as a function of the diet’s nutrient composition.

Seo et al. (2021) conducted a meta-analysis using 306 means from 69 studies to evaluate the accuracy of a no-intercept linear equation to describe the relationship between DE and ME. Additionally, using the study as a random variable, equations to predict the ME:DE ratios were developed for growing and finishing beef cattle, and the y-intercept did not differ from zero. Excluding the intercept from the equation more appropriately represented the relationship between DE and ME based on Akaike and Bayesian information criteria than equations using a y-intercept (Seo et al., 2021). Therefore, in the Seo et al. (2021) analysis, the ME:DE ratio was predicted as 0.9410 + 0.0042 × DMI (kg) – 0.0017 × NDF (% DM) – 0.0022 × CP (% DM) based on dietary components. Although the model accuracy was high (CCC > 95%) and the RMSPE was less than 5% of the observed mean, predicting a ratio may be problematic. Depending on ingredient composition, the proportion of urinary and methane energy loss is not necessarily consistent across diets, so that the predicted ratio could be inaccurate if this inconsistency is not modeled effectively by DMI and the two dietary components used (NDF and CP) in their equation. Our current results suggest that the ratio would increase with increasing DE concentration, consistent with data noted previously for higher-concentrate diets, and consistent with expected changes in methane and urinary losses as DE concentration increases. In the instances of intakes near maintenance or a negative energy balance, the quantity of urinary nitrogen lost could be increased, which might affect the accuracy in predicting the ratio. Previous research indicates that as intake increases from 1- to 2-times maintenance, the ratio of ME:DE is increased by 7.5% (Blaxter and Wainman, 1964). Likewise, Vermorel and Bickel (1980) reported that as the level of DMI increased, the ME:DE ratio increased in growing lambs fed chopped or pelleted hay diets. Others have reported an increase in the ME:DE ratio of approximately 5% to 6% as the level of DMI increased from 1- to 2-times maintenance when feeding high-concentrate finishing diets based on steam-flaked or dry-rolled corn (Hales et al., 2012, 2013; Jennings et al., 2018).

Hales et al. (2013) fed growing steers diets with increasing concentrations of wet distillers grains plus solubles from 0% to 45% of DM that replaced steam-flaked corn and a portion of yellow grease and urea. The CP content of the diets increased from 13.3% to 20.2% of diet DM as wet distillers grains plus solubles inclusion rate increased, leading to a linear increase in urinary energy as byproduct inclusion increased in the diet. Likewise, methane energy loss increased linearly as the wet distillers grains plus solubles concentration was increased in the diet; however, although both were linear responses, the rate of the increase for urine energy loss and methane energy loss differed. The slope for methane energy loss was 44% greater than the slope for urinary energy loss, indicating that the relationship between the two variables is inconsistent across diets.

Most NE_m_ and NE_g_ values for feed ingredients are calculated from ME using the cubic equations proposed by Garrett (1980), which were derived by converting DE to ME with a constant of 0.82. The manner in which ME was measured in some of the original California Net Energy System (**CNES**; Lofgreen and Garrett, 1968) studies is not clear, which was discussed by Galyean et al. (2016). Because the NE_m_ requirement in the CNES was calculated by regression of log heat production on ME intake, using a new equation to calculate ME concentration could affect the estimate of the NE_m_ requirement. In contrast, NE_g_ in the CNES was estimated from carcass specific gravity using equations reported by Kraybill et al. (1952) and Reid et al. (1955) to calculate body composition from the caloric values of fat and protein and thereby not affected by the DE-to-ME conversion factor. Using the original CNES database, Galyean et al. (2016) recalculated the ME values from their equation for the conversion of DE to ME (ME=0.9611×DE−0.2999) and compared estimates of the NE_m_ requirement for the recalculated vs. original data. The NE_m_ requirement (77 Mcal/kg BW^0.75^) did not differ between the recalculated and original CNES data; thus, Galyean et al. (2016) adjusted the cubic equations of Garrett (1980) to ensure that the estimates of NE_m_ and NE_g_ concentration resulting from the use of their DE-to-ME conversion equation would yield estimates equal to the current values in NASEM (2016). We recommend using the cubic equations reported by Galyean et al. (2016) for calculating dietary NE_m_ and NE_g_ values when the ME is estimated from equation 2.

Although DE and ME are highly dependent on dietary nutrient composition, there are likely host effects that contribute to differences in the conversion of DE to ME, especially methane production. Host genotype explained 24% of the variation in methane production by 750 dairy cows (Zhang et al., 2020). Beef cattle typically consume less DM than dairy cows, and albeit a lesser energetic loss in cattle compared with fecal losses and heat production, methane production generally accounts for 2.5% to 12% of energy lost (Johnson and Johnson, 1995), as a proportion of energy consumed. If the host genotype can account for nearly one-quarter of the variation in methane production, predicting ME solely from dietary attributes could underestimate or overestimate ME when uncommon genotypes are evaluated. Indeed, if the genotype is known, it could be used in addition to DMI and improve the ability to predict methane.

### Predicting methane from DMI

From a biological standpoint, equation 1 suggests that across the broad range of DE concentrations in our database (1.76 to 3.88 Mcal/kg of DMI), the combined energy lost as urine and methane (Mcal) is relatively constant per kilogram of DMI. Given that the proportions of methane and urine were known in our updated dataset, by fitting a model with no slope but an adjustment for random intercepts associated with studies, we derived a citation-adjusted value for the energy (Mcal/kg of DMI) lost as methane. If this value is multiplied by daily DMI, it will yield an estimated energy lost as methane (Mcal). Thus, our first proposed equation to predict energy lost as methane is:


$$\begin{array}{l} CH_{4},\;Mcal/d=0.2433\times DMI,\;kg/d; \end{array}$$ (3)

where SE = 0.0134, with 95% confidence limits of 0.216 and 0.271 on the coefficient.

Recognizing that methane production decreases per unit of intake energy as intake increases above maintenance (NASEM, 2016), we further examined the relationship in our database between multiples of maintenance intake and daily energy lost as methane (Mcal/kg of DM). The resulting equation, adjusted for slope and intercept effects of study, was:


$$\begin{array}{ll} CH_{4},\;Mcal/kg\;DMI= & \;0.3344-0.05639\\ & \times multiple\;of\;maintenance\; \end{array}$$ (4)

where multiple of maintenance is Mcal of NE_m_ intake divided by Mcal of NE_m_ required, and *r*^2^ = 0.536, RMSPE = 0.0245, and *P* < 0.001 for the intercept and slope; 95% CIs: intercept [0.273, 0.396] and slope [−0.0957, −0.0171]. The value derived from equation 4 would then be multiplied by DMI (kg/d) to yield an estimate of daily methane production (Mcal). For both equations 3 and 4, the daily methane production expressed in Mcal/d was converted to g/d using conversion factors of 9.45 kcal/L and 0.716 g/L for methane.

Using the second, independent literature database described previously, we evaluated the accuracy and precision of predicting methane using equations 3 and 4 as well as four equations in the literature. We focused comparisons on equations from the literature that used DMI or intake energy to predict methane and were thereby similar in approach to equations 3 and 4. Thus, equations selected for the comparison were among those evaluated by van Lingen et al. (2019), which included their DMI_C equation, the IPCC Tier 2 equation for higher-forage diets, the Global Network Tier 2 equation, and the Combined equation 2c from Ellis et al. (2007). When an equation specified intake energy or methane production in MJ/d, a conversion of 4.184 was used to convert MJ to Mcal. Specific equations used from these publications were as follows:

1. IPCC Tier 2 (equation 9 in van Lingen et al., 2019; higher-forage diets):

$$\begin{array}{l} CH_{4},\;g/d=intake\;energy,\;MJ/d\times 0.065÷0.05565; \end{array}$$

2. Ellis et al. (2007; combined equation 2c):

$$\begin{array}{l} CH_{4},\;MJ/d=3.27+0.74\times DMI,\;kg/d; \end{array}$$

3. van Lingen et al. (2019; equation 1 – DMI_C):

$$\begin{array}{l} CH_{4},\;g/d=54.2+12.6\times DMI,\;kg/d; \end{array}$$

4. Global Network Tier 2 (equation 8 in van Lingen et al., 2019):

$$\begin{array}{l} CH_{4},\;g/d=0.061\times intake\;energy,\;MJ/d÷0.05565 \end{array}$$

**Figure 2.**
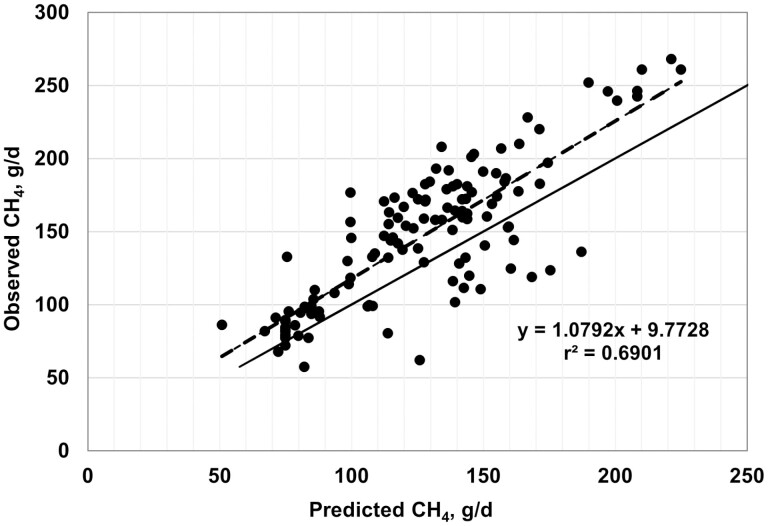
Plot of observed vs. predicted methane (g/d) using equation 3 developed in this study. The solid line indicates y = x, and the dashed line depicts the fitted regression (95% confidence limits: intercept −6.7137, 26.2592; slope = 0.9522, 1.2062).

**Figure 3.**
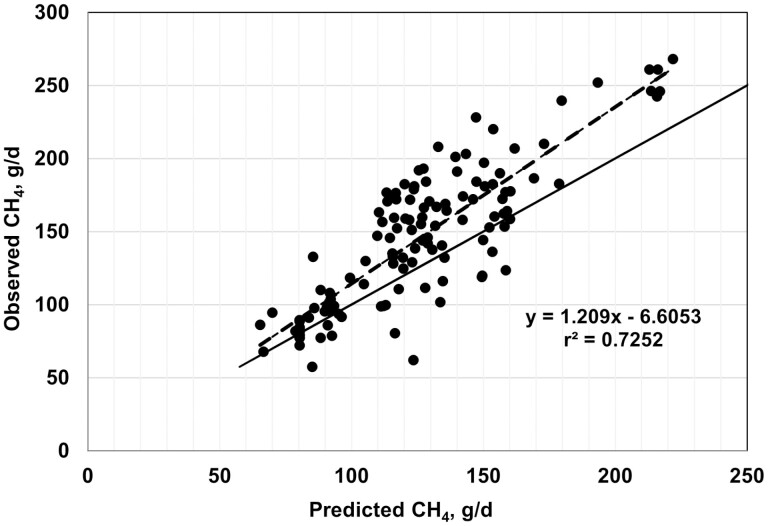
Plot of observed vs. predicted methane (g/d) using equation 4 developed in this study. The solid line indicates y = x, and the dashed line depicts the fitted regression (95% confidence limits: intercept −23.4790, 10.2685; slope = 1.0783, 1.3397).

**Figure 4.**
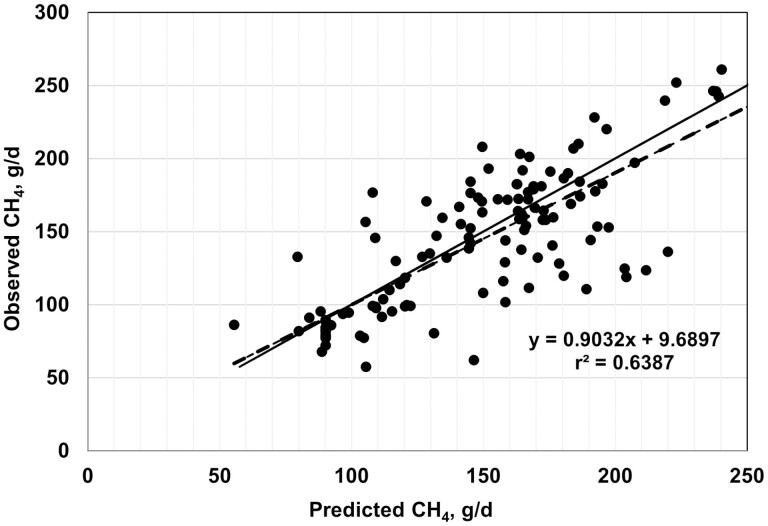
Plot of observed vs. predicted methane (g/d) using the IPCC Tier 2 equation (equation 9 in van Lingen et al., 2019). The solid line indicates y = x, and the dashed line depicts the fitted regression (95% confidence limits: intercept −8.7661, 28.1456; slope = 0.7839, 1.0224).

**Figure 5.**
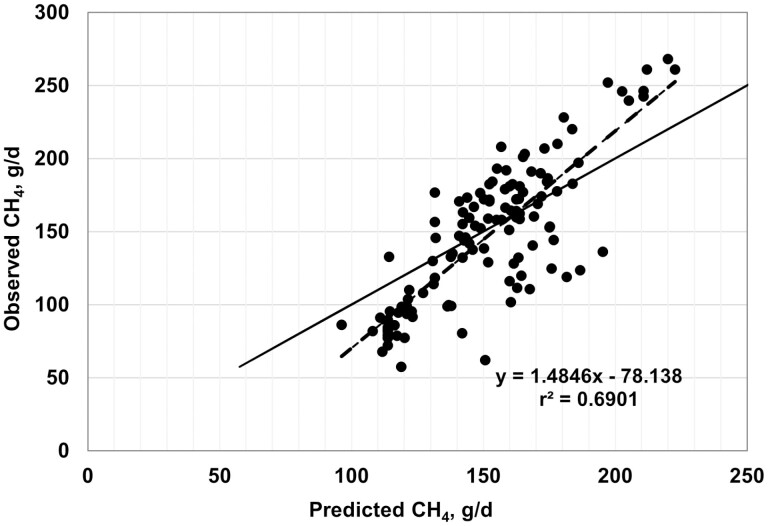
Plot of observed vs. predicted methane (g/d) using the DMI equation (equation 2c in Ellis et al., 2007). The solid line indicates y = x, and the dashed line depicts the fitted regression (95% confidence limits: intercept −104.6951, −51.5780; slope = 1.3099, 1.6593).

**Figure 6.**
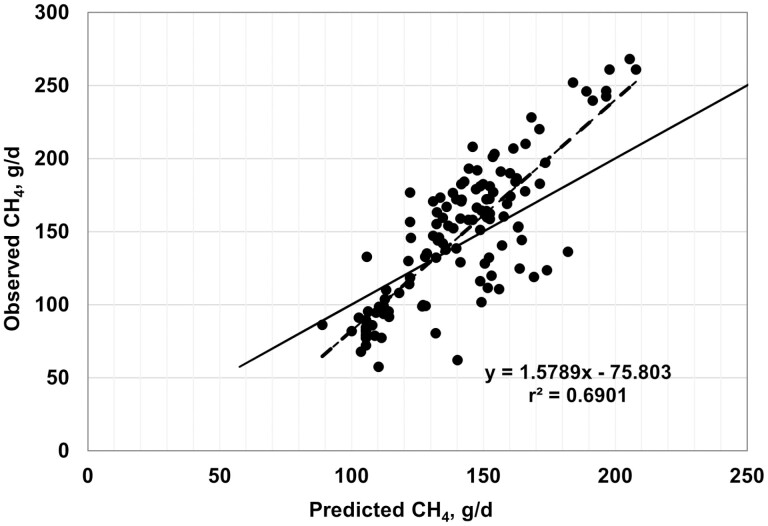
Plot of observed vs. predicted methane (g/d) using the DMI equation (DMI_C equation 1 in van Lingen et al., 2019). The solid line indicates y = x, and the dashed line depicts the fitted regression (95% confidence limits: intercept −102.0908, −49.5159; slope = 1.3931, 1.7647).

**Figure 7.**
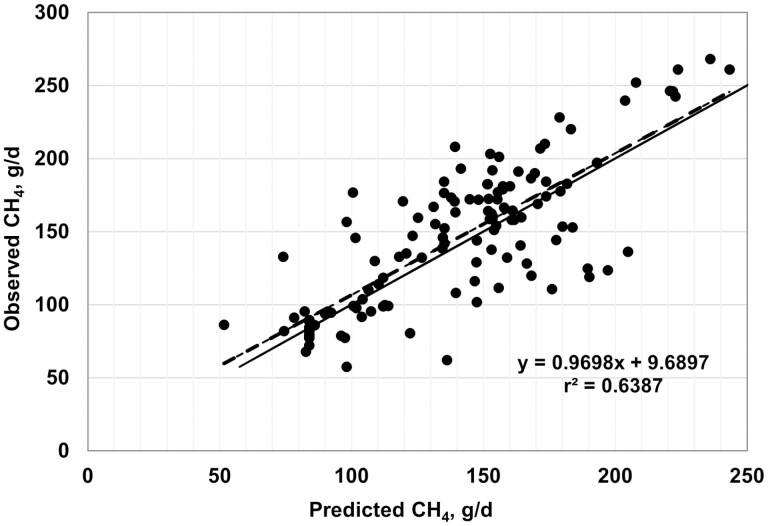
Plot of observed vs. predicted methane (g/d) using the Global Network Tier 2 equation (equation 8 in van Lingen et al., 2019). The solid line indicates y = x, and the dashed line depicts the fitted regression (95% confidence limits: intercept −8.76612, 28.14557; slope = 0.84176, 1.09794).

Plots of observed vs. predicted values are shown in Figures 2–7, and equation performance statistics are presented in Table 2. The coefficient of determination was similar among the six equations evaluated, ranging from 0.639 to 0.725. Equations 3 and 4 were in the upper end of the range with *r*^*2*^ values of 0.690 and 0.725, respectively. Despite the greater *r*^2^ values for equations 3 and 4 compared with the other equations, the RMSPE was greater for these two equations than for the other four equations from the literature (33.7 and 32.9 g/d, respectively, vs. an average of 30.5 g/d for the other four equations; Table 2). Decomposition of the RSMPE indicated that mean bias was substantially greater for equations 3 and 4 than for the four extant equations tested (34% and 34.8% of RMSPE, respectively, vs. an average of 2.9% for the other four equations). Conversely, slope bias for equation 3 was among the least of the six equations (0.8% of the RMSPE), with an increase to 4.8% slope bias for equation 4. The IPCC Tier 2 and Global Network Tier 2 equations also had low slope bias (1.9% and 0.2%, respectively), whereas the Ellis et al. (2007) and van Lingen et al. (2019) equations had much greater slope bias (18.5% and 22.5%, respectively) than the other four equations. For all six equations, random errors accounted for the majority of the RSMPE, but error bias was the least for equations 3 and 4 (average of 62.9% vs. 86.3%), reflecting the greater mean bias for these equations. The CCC ranged from 0.68 to 0.78 over the six equations, with equations 3 and 4 at a CCC of 0.73. Among the six equations, only equation 3, the IPCC Tier 2, and the Global Network Tier 2 equations had 95% confidence limits for the intercept and slope of the observed vs. predicted plots that included 0 and 1, respectively (Figures 2–7). Thus, none of the six equations we evaluated showed the degree of agreement between observed and predicted values that would be desirable for the most accurate predictions of methane emissions.

**Table 2.** Equation performance statistics for the two equations developed to predict daily methane emission in the current study (equations 3 and 4) compared with extant prediction equations in the literature

Equation source	*r* ^2^	RMSPE^1^, g/d	RMSPE, % of mean	% of RMSPE			CCC^2^
				Mean bias	Slope bias	Error bias	
Equation 3	0.690	33.7	23.4	34.0	0.8	65.3	0.73
Equation 4	0.725	32.9	22.9	34.8	4.8	60.5	0.73
IPCC Tier 2^3^	0.639	30.0	20.9	2.5	1.9	95.6	0.79
Ellis et al. (2007) ^4^	0.690	30.8	21.4	3.4	18.5	78.1	0.70
van Lingen et al. (2019) ^5^	0.690	31.4	21.8	2.3	22.5	75.2	0.68
Global Network Tier 2^6^	0.639	29.9	20.8	3.4	0.2	96.4	0.78

RMSPE, root mean square prediction error.

CCC, concordance correlation coefficient.

Equation 9 in van Lingen et al. (2019).

Equation 2c in Ellis et al. (2007).

Equation 1 (DMI_C) in van Lingen et al. (2019).

Equation 8 in van Lingen et al. (2019).

All six of the equations we evaluated effectively use DMI to predict methane emissions. For the two equations that use GE intake, DMI would be the major driver of the estimate, as GE concentrations do not vary greatly across various types of common energy feedstuffs, although GE will be greater in high protein or high-fat feeds. It is commonplace for DMI to be used to predict methane emissions from cattle (Reynolds et al., 2011; Hristov et al., 2013; NASEM, 2016). As cattle consume more dry matter (**DM**), more methane is produced because of greater substrate for microbial fermentation. Indeed, DMI has the greatest effect on methane production among various prediction models (Niu et al., 2018). Yan et al. (2000, 2009) reported that if DMI is omitted from the model, the model will underpredict methane production when DMI is low and overpredict methane production when DMI is high. From a practical standpoint, DMI for pen-fed cattle is an attractive variable to use in predicting methane because it is frequently known or can be estimated with a relatively high degree of accuracy.

The greater *r*^2^ for equation 4 vs. equation 3 suggests that the adjustment of intake above maintenance slightly increased the variation in observed methane production accounted for by the prediction equation. Similarly, equation 4 had a somewhat lesser RMSPE than equation 3, but mean bias and slope bias were greater with equation 4 vs. equation 3. As a proportion of intake energy, methane production decreases as energy intake increases, which explains why it can be over- or underpredicted as the level of intake changes (Blaxter and Wainman, 1964; Blaxter and Clapperton, 1965; Hales, 2019). Increased DMI can increase the passage rate, shorten ruminal retention time, decrease digestibility, and thereby decrease methane production scaled to DMI (Boadi et al., 2004). Methane production per unit of intake decreases with increasing DMI, suggesting a greater ruminal turnover rate resulting in decreased ruminal digestibility of the diet (Buddle et al., 2011). As DMI increases from 1- to 2-times maintenance, methane energy lost per unit DMI can be decreased by up to 43% (Hales et al., 2013). Predicting methane production from multiples of maintenance could be useful because it includes a combination of DMI, BW, and net energy required for maintenance. Nonetheless, the extent of improvement in predictions for equation 4 compared with equation 3 was small. More research is needed to determine whether this correction would be more important in datasets with a greater variation in energy intake above maintenance than was the case in our development dataset and how dietary composition might affect results.

The equal or greater *r*^2^ and relatively low slope bias for equations 3 and 4 suggest that using a constant megacalories per kilogram of DMI value for methane could be an effective means of predicting the emission of methane for diets similar to those in our dataset. Nonetheless, the much more significant mean bias for our two equations than for the literature equations suggests that the coefficient used in equation 3 (and functionally in equation 4) needs adjustment. Specifically, because the predicted values were consistently less than observed values, the coefficient is likely less than it should have been to yield an accurate prediction. This is not surprising, as the average Mcal of methane produced per kilogram DMI in the evaluation dataset averaged 0.2841 vs. a citation-adjusted mean of 0.2433 (0.2475 for the unadjusted mean) in the development dataset. This difference could reflect variation in diet composition, DMI, and BW of the animals used in the two datasets, as well as other unknown factors. For example, the average BW of cattle used in the development dataset was approximately 363 kg vs. approximately 400 kg in the evaluation dataset. Similarly, DMI per unit of BW was less in the development dataset than in the evaluation dataset (1.67% vs. 2.13% of BW). As noted previously, BW can affect DMI and the rate of passage, both of which can influence methane production (Hristov et al., 2013; Niu et al., 2018). Cattle with lighter BW have less total DMI and thus produce less methane.

One option to address the mean bias problem from equations 3 and 4 is to correct the value of the coefficient. Indeed, merely using the upper bound of the 95% confidence limit for the coefficient (0.271) would eliminate most of the mean bias. Although this approach might result in greater agreement between observed and predicted values for our evaluation dataset, it would not necessarily work for other independent datasets that had different dietary and animal characteristics. A practical approach could be to develop coefficients for specific diet types such as high-forage vs. high-starch, levels of DMI relative to BW, multiples of maintenance energy intake, feed processing, or other production variables that affect the emission of methane. In our updated development database, if the data are sorted by energy lost as methane (Mcal/kg of DM), the top 50% of methane production values (average methane = 0.315 Mcal/kg of DM) had lower mean starch (16.7% of DM), greater NDF (47.5% of DM), and lower EE (3.4%) concentrations in the diet than the bottom 50% of values (average methane = 0.18 Mcal/kg of DM; starch = 37.5% of DM; NDF = 28.2% of DM; EE = 4.7% of DM). As noted above for comparing the development and evaluation datasets, differences in DMI per unit BW or other dietary components also could change the methane coefficient. Further evaluation of methane datasets to parse out groupings of data might prove fruitful in yielding a small number of coefficients that could provide a practical means of predicting methane emissions over a broad range of diets and feeding conditions.

Based on the presently available data, we recommend adopting the equation ME = DE – 0.39, where ME and DE are expressed in Mcal/kg of DM. The methane equations we developed and evaluated might have practical utility, but as with most equations based on DMI or intake energy, more research is necessary to improve the relationship between observed and predicted values. Methane production coefficients for specific diet types, levels of DMI relative to BW, or other variables that affect the emission of methane should be investigated to predict methane emissions of beef cattle accurately.

## Supplementary Material

skac013_suppl_Supplementary_Data

## Abbreviations

ADF: acid detergent fiber;
BW: body weight
CCC: concordance correlation coefficient
CNES: California Net Energy System
CP: crude protein
DE: digestible energy
DM: dry matter
DMI: dry matter intake
EE: ether extract
GE: gross energy
ME: metabolizable energy
MSPE: mean squared prediction error
NDF: neutral detergent fiber
NE_m_: net energy for maintenance
NE_g_: retained energy
NRC: National Research Council
RMSPE: root mean square prediction error
